# Hierarchical status is rapidly assessed from behaviourally dominant faces

**DOI:** 10.3758/s13415-023-01108-1

**Published:** 2023-05-17

**Authors:** Alan J. Pegna, David Framorando, Zhou Yu, Zak Buhmann, Nicole Nelson, Barnaby J. W. Dixson

**Affiliations:** 1 School of Psychology, The University of Queensland, Saint Lucia, Brisbane, QLD Australia; 2 School of Psychology, University of Adelaide, Adelaide, SA Australia; 3 School of Health and Behavioural Sciences, University of the Sunshine Coast, QLD Sippy Downs, Australia; 4 Faculty of Psychology and Educational Science, University of Geneva, Geneva, Switzerland

**Keywords:** Face, N170, VPP, P2, LPP, Dominance, Social interaction, Emotion

## Abstract

Recognition of social hierarchy is a key feature that helps us navigate through our complex social environment. Neuroimaging studies have identified brain structures involved in the processing of hierarchical stimuli, but the precise temporal dynamics of brain activity associated with such processing remains largely unknown. In this investigation, we used event-related potentials (ERPs) to examine the effect of social hierarchy on the neural responses elicited by dominant and nondominant faces. Participants played a game where they were led to believe that they were middle-rank players, responding alongside other alleged players, whom they perceived as higher or lower-ranking. ERPs were examined in response to dominant and nondominant faces, and low-resolution electromagnetic tomography (LORETA) was used to identify the implicated brain areas. The results revealed that the amplitude of the N170 component was enhanced for faces of dominant individuals, showing that hierarchy influences the early stages of face processing. A later component, the late positive potential (LPP) appearing between 350–700 ms, also was enhanced for faces of higher-ranking players. Source localisation suggested that the early modulation was due to an enhanced response in limbic regions. These findings provide electrophysiological evidence for enhanced early visual processing of socially dominant faces.

## Introduction

Hierarchy and the pursuit of social status defines the evolution of human social organisations, wherein social rank determines access to resources, influential social partners, and mates (Cheng et al., 2013; von Rueden & Jaeggi, 2016; von Rueden et al., 2019). An individual’s standing within their social hierarchy also is reflected in their health; lower rank negatively impacts well-being, stress, morbidity, and survival (Boyce, 2004; Sapolsky, 2004, 2005).

Hierarchies exist in all social mammals, and in humans they are ubiquitous across cultures and ages. They have been observed even in the most egalitarian hunter-gatherer societies (Boehm, 1999) and in groups of children as young as 2 years old (Boyce, 2004; Strayer & Strayer, 1976). Because of the relevance of hierarchical information, individuals appear to be very adept at detecting dominance even when little information is available. Research has shown that dominance and submissiveness are judged automatically (Moors & De Houwer, 2005) and that dominance is recognised even in the absence of social context (Oosterhof & Todorov, 2008) and across different cultures (Jones et al., 2021).

Dominance triggers increased visual attention (Foulsham et al., 2010; Maner et al., 2008) and memory processes (Ratcliff et al., 2011). Along these lines, fMRI has showed that the viewing of high-status faces leads to increased activity in brain areas associated with visual and attentional processing compared with low status faces (Farrow et al., 2011; Ligneul et al., 2017; Qu et al., 2017; Zink et al., 2008). For example, Zink et al. (2008) manipulated perceived social rank during an interactive game, whereas BOLD brain activation patterns were examined. When viewing dominant players, strong responses in parieto-occipital, amygdala, and prefrontal areas were elicited, suggesting that individuals perceived as hierarchically dominant activated perceptual, attentional, and cognitive systems. Based on these results, Breton et al. (2014) later suggested that the enhanced visual processing of dominant faces might be triggered by an early amygdala response for dominance. Specifically, the authors proposed that social dominance might be processed via a fast subcortical pathway to the amygdala, which is known to respond to highly sensitive, emotional stimuli (Öhman, 2005) and that amygdala activation should in turn activate visual and higher-level areas via a feedback mechanism.

Unfortunately, imaging techniques lack high temporal resolution and cannot establish the timing of such an effect. It is therefore impossible to establish, by using these methods, whether processing occurs in the early face processing stages or whether the perception of social dominance relies on later, more complex cognitive processing.

Surprisingly few studies have investigated the time course of brain activation in response to dominant faces, using methods with a high temporal resolution, such as electro-encephalography (EEG). Those that have been performed have explored the response to faces differing in trustworthiness and dominance as determined by facial characteristics and have reported changes in brain activation at the very early stages of neural processing (i.e., within 200 ms) (Marzi et al., 2014). Such rapid effects thus point to uncontrolled and unintentional processes, probably occurring automatically and without awareness. A few other investigations have studied faces whose dominance was determined on the basis of competitive games or advance information. Two of these reported an early response (Feng et al., 2015; Santamaría-García et al., 2015), whereas two others have reported only late effects, arising after 400 ms (Breton et al., 2014, 2019). Such differences are likely linked to differences in experimental procedures, as pointed out by Breton et al. (2019).

Studies that reported early effects showed that dominance elicited difference on the P1 and N170 components, which reflect respectively the allocation of attentional resources and the encoding of facial stimuli (Bentin et al., 1996; Hopf & Mangun, 2000). Such results provide insights into neural components underpinning face perceptions.

Thus, the sum of current findings suggest that face dominance might be processed in the early perceptual stages, or during later periods (i.e., >300 ms), when more complex cognitive computations are performed. Moreover, some authors suggest that the modulation of early visual and later ERPs may be triggered by greater activity of the amygdala, which modulates visual and cognitive processing, although this possibility remains uncertain.

In the present study, we therefore investigated whether the viewing of dominant and nondominant faces, determined on the basis of an interactive game, modulated early or late neural responses. Source localization was further performed on the periods of differential activation to establish the areas involved during these periods and identify the dynamics of neural activation for dominance. To induce the perception of social rank, participants completed a competitive game consisting of a reaction time task played against (fictitious) players. They were ranked repeatedly as 2-star players, while their hypothetical counterparts were ranked either as 1-star (inferior) or 3-star (superior) players. We hypothesised that the N170, sensitive to faces (Bentin et al., 1996; Batty & Taylor, 2003) but also emotional expression (Blau et al., 2007; Pegna et al., 2008) would be modulated by behavioural dominance, with the strongest effects occurring when responding to the faces of the socially dominant players. Second, we hypothesised that the underlying neural generators would indicate enhanced visual extrastriate activity, which would potentially be attributable to a heightened amygdala response (Framorando et al., 2021), in this case as a result of perceived social dominance.

## Materials and methods

### Participants

Twenty-eight participants from the University of Queensland took part in the study (10 males; mean age: 23.4 ± 8.2 years). All participants were right-handed and had normal or corrected-to-normal vision. None reported a history of neurological or psychiatric disorder. The study was approved by the Ethics Committee of the University of Queensland. Participants gave their informed consent before initiating the experiment and received either 20 AUD or 2 course credits for their time.

### Stimuli

The stimuli were composed of greyscale, front-view photographs of four male and four female neutral faces selected from the Karolinska Directed Emotional Faces Database (Goeleven et al., 2008). Participants were only presented with faces of their gender during the task. Participants also were photographed before the experiment on a grey background, and their greyscale photograph was adjusted and cropped to the same dimensions as the experimental faces used in the procedure.

### Design

Social hierarchy was induced by comparing the participant’s performance to the performance of other players in a computerised game similar to that reported elsewhere (Santamaría-García et al., 2015; Zink et al., 2008). In the game, participants completed a reaction-time task and received what they believed was feedback on their performance. Alongside this information, indications were provided regarding the scores of four players, whom participants were led to believe were students who had performed the experiment on previous days.

The game consisted in a reaction time (RT) task in which participants were required to respond as rapidly when a centrally presented disk changed colours from blue to green. Participants were asked to press the space bar on the keyboard immediately after the colour change. They were told that if their RT was within a short, preestablished threshold (that they were not informed of), they would “win” the round and be awarded one virtual dollar. However, if they failed to respond within the given period, they would “lose” the round and would not receive the reward. Participants were further told that throughout the game, their performances would be compared and ranked with those of 4 other players who had participated in the experiment on previous days. They were told that the performances of these alleged players were only provided as a benchmark for them to judge their own performance and that this was not to be considered a competition.

Two of the reference players were presented as winning only 10-20% of the trials and thus were staged as inferior-performing players. The other two faces were shown to have a winning rate of 80-90% and were thus presented as superior players. Unbeknownst to the participants, feedback regarding their own performance was assigned a win or lose value with a 50% probability. Participants were therefore always ranked as “intermediate.” In order to limit any detection of this manipulation, two constraints were imposed: any RT below 200 ms by the participant would necessarily indicate a “win,” and any RT longer than 750 ms would necessarily give rise to a “lose.” Lastly, to protect against inattentive participants, the experiment was automatically paused if three trials showed RTs >750 ms, and participants were informed that their responses were too slow before resuming.

**Fig. 1:**
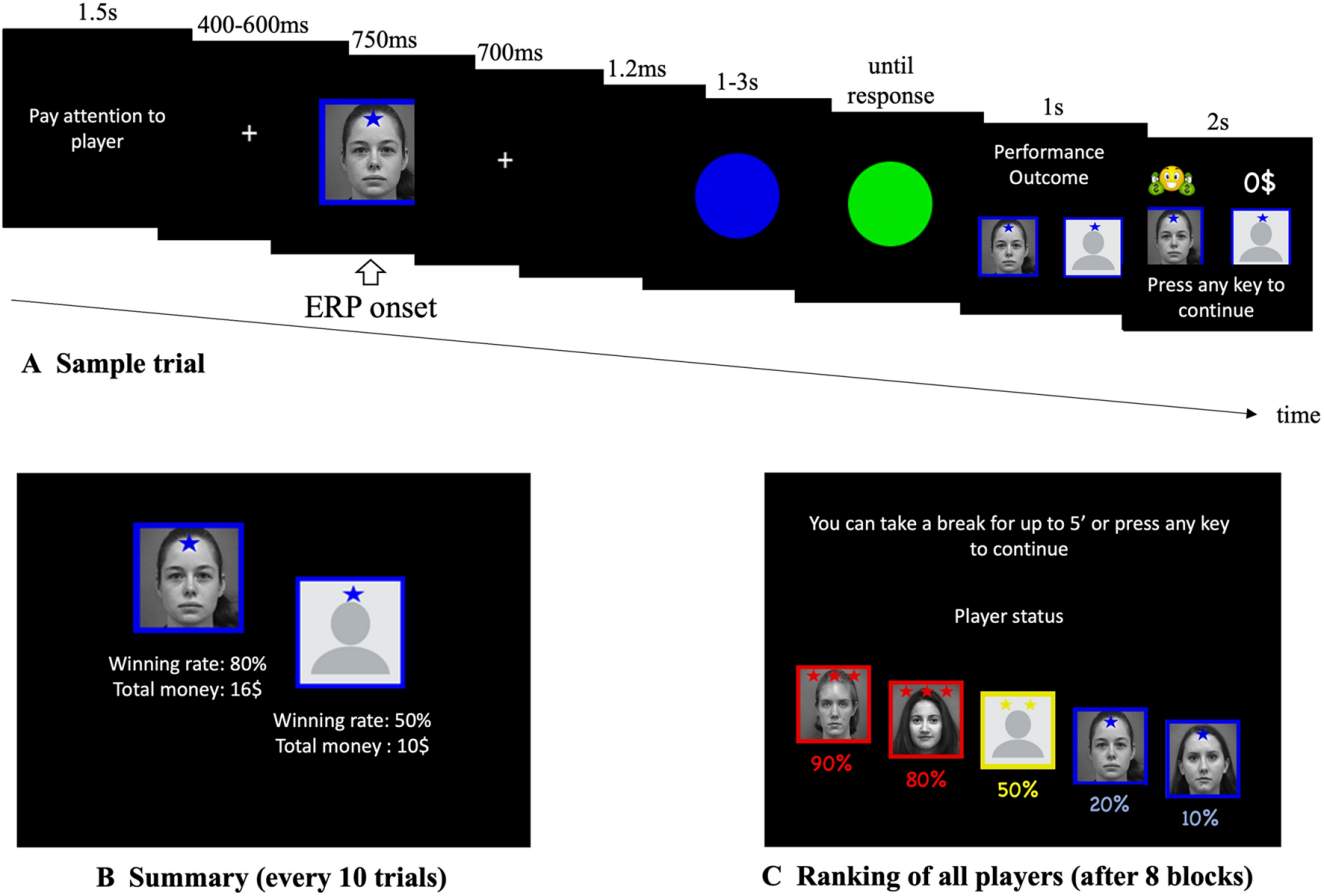
(**A**) Examples of a typical trial (stimuli are not shown to scale). (**B**) After 10 trials with the same player, feedback was provided regarding said player and the participant with indications of hierarchical status (inferior = 1 star; superior = 3 stars). All players received 1 star for the first two blocks. Later, players were updated with participants always receiving an intermediate (2-star) rating. (**C**) After the first two blocks, a global ranking was provided that was then used throughout the subsequent blocks

Perceived hierarchy was created using a star-ranking system. Faces were presented with their star-ranking throughout the procedure by framing the faces in blue (RGB: 0, 0, 230) or red (RGB: 230, 0, 0), and superimposing their star ranking wherein a single star reflected an inferior player and 3 stars (superior player), in blue or red on the forehead of the face (Fig. 1). The participants’ intermediate level was indicated by a yellow frame and two yellow stars (RGB: 230, 230, 0). All depictions of facially dominant and non-dominant conspecifics were counterbalanced, as was the attribution of red and blue colours for the framing and stars.

### Procedure

Trials began with a prompt asking the participant to pay attention to the face of the reference player (1.5 s) with whom their performance would be compared in that trial. This was followed by a fixation cross (random duration: 400-600 ms) and then by a photograph of the reference player (6 cm x 7 cm) for 750 ms. A fixation cross was then presented again (700 ms) followed by a blank screen (1.2 s) and then the target blue disc (4-cm diameter). After a random duration of between 1,000 and 3,000 ms, the blue target changed to green, necessitating the participant’s response. After the response, a blank screen appeared for 1,000 ms followed by the feedback slide appeared (2,000 ms) showing the photographs of the participant and the reference player side by side, with an indication on whether each had won or lost in that particular trial (self-paced with a maximum time of 4 s; Fig. 1). A “win” was indicated by a smiling emoji holding a wad of money on top of the face. A “lose” situation was indicated with “$0.” Because the procedure was not competitive, both players could win or lose in the same trial. The same reference player was shown throughout each block of ten trials. After this block, a new feedback slide was presented showing the total amount of money earned by the participant and the alleged reference player (4,000 ms), and the star ranking was updated accordingly. The next reference player was then presented for the ten following trails. Two blocks were performed showing each of the four reference players and the participant as 1-star players (2 blocks x 4 players x 10 trials).

For reasons of plausibility, alleged players and participants were ranked as 1-star in the first eight blocks (i.e., the first 2 blocks with each of the alleged players). After the eighth block, and for the remaining 16 blocks (i.e., 4 blocks with each of the 4 players), the rankings of the players were updated according to the supposed performance. At this point, two players became 3-star (dominant) players, the participant became a 2-star (intermediate) player, and the remaining two alleged players kept their 1-star ranking (inferior player).

The experimental procedure was run in a dark room using E-Prime 3.0 (Psychology Software Tools, Pittsburgh, PA). Stimuli were presented on a 24” LCD (ASUS resolution: 1,080 x 1,920 pixels; refresh rate: 60 Hz), on a screen situated 60 cm from the participant’s eyes. Participants also were instructed to pay attention to the face of the alleged player. To ensure that the participants were attending the faces of the reference player at the onset of each trial, a small red dot appeared randomly on the forehead of the face on 10% of trials. Participants were required to respond by immediately pressing on the space bar when the red dot was present. These target faces were excluded from further analysis.

After the experiment, each participant was given a 7-point Likert scale questionnaire containing ten questions, asking about the participants’ feelings caused by the view of the alleged participants (anxious, happy, motivated) and the perceived influence that the reference players had on their performance. Participants were asked if they believed the reference players were real, and if they believed in the feedback provided to them regarding performance.

### EEG recording

Continuous EEG was acquired at 1,024 Hz by using the Biosemi ActiveTwo system (Biosemi, Amsterdam, The Netherlands) with 64 equally spaced scalp electrodes placed according to the international 10-20 system. Two additional electrodes (active CMS: common mode sense and passive DRL: driven right leg) were used as reference and ground to compose a feedback loop for amplifier reference. Two external electrodes EOG were used to monitor eye blinks and saccades. One was placed on the outer canthus of the right eye and one above the right eyebrow. Brain Vision Analyzer 2.1 (Brain Products, Gilching Germany) was used for EEG analysis.

### ERP processing

Epochs, time-locked to the onset of the face, were established from 100 ms before to 500 ms after stimulus onset. This duration was chosen to minimize the number of epoch rejections due to eye-blinks, which were frequently observed after ~500 ms. Traces were visually inspected and those containing eye movements, blinks, and electrical artefacts were excluded from further analysis. Noisy electrodes or those producing many artefacts were removed and re-interpolated using 3D splines. An ERP was then computed for the faces presented in the first 2 blocks (control condition in which all players and the participant were ranked as 1-star). Two other ERPs were computed for the subsequent 4 blocks: one for the dominant and one for the nondominant faces. ERPs were baseline corrected by using the 100 ms prestimulus period. EEG was bandpass filtered between 0.1 and 30 Hz (24dB/octave), and the signal was re-referenced to the average reference.

**Fig. 2:**
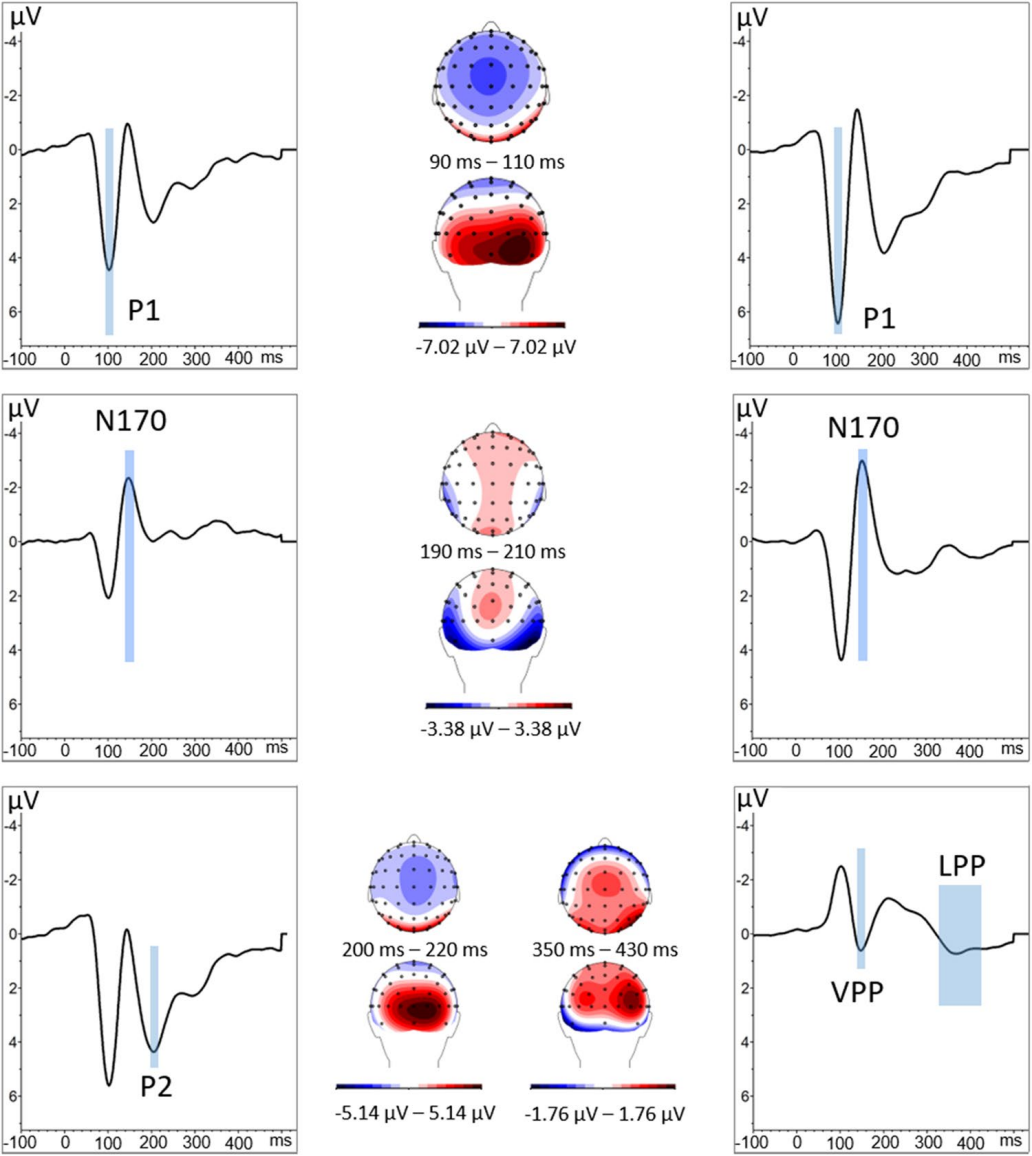
Grand-average waveform for the P1 (O1/O2, PO7/PO8, P9/P10), N170 (P7/P8, P9/P10 and TP9/TP10), P2 (Iz/O1/O2/Oz/PO7/PO8), and VPP and LPP (C1, C2, Cz, CP1, CP2, CPz, FC1, FC2, FCz) averaged over the electrodes of interest. Traces are expressed as mV over time (in ms). Centre: Scalp topographies viewed from above and behind for the mean during the respective time periods with corresponding color code below. The time window and scalp topography for the VPP is the same as for the N170 (central insert)

The individual ERPs obtained for the three conditions were then used to calculate three grand average ERPs (one for each condition), as well as an overall grand mean, which included all conditions. Statistical analyses were performed on the mean amplitudes of the P1, N170, VPP (considered to be the positive counterpart of the N170; Joyce & Rossion, 2005), P2, and LPP. These were measured at their maximal occurrence on the grand mean ERPs computed over all conditions (Fig. 2) and were observed in the following time windows: 90-110 ms, 145-165 ms, 200-220 ms, and 350-430 ms. Electrodes of interest were selected, because they showed maximum activity and were consistent with those habitually used to investigate these components (O1/O2, PO7/PO8, P9/P10 for the P1; P7/P8, P9/P10 and TP9/TP10 for the N170; Iz/O1/O2/Oz/PO7/PO8 for P2 and C1, C2, Cz, CP1, CP2, CPz, FC1, FC2, FCz for the LPP and the VPP).

### Statistical analyses

An analysis of covariance was carried out on the P1, N170, P2, and LPP amplitudes using Dominance Category (Dominant, Nondominant, Control) and Electrode Hemisphere (Left vs. Right) as factors. Violations of sphericity and p-values were corrected according to the epsilon of Greenhouse-Geisser or Huynh-Feldt. In line with Field (2013), the Huynh-Feldt correction was preferred when the Greenhouse-Geisser estimate of sphericity (ε) was >0.75, whereas Greenhouse-Geisser was used for ε <0.75.

### Source localization

Source estimation was conducted by using Low-Resolution Electromagnetic Tomography (LORETA, Pascual-Marqui et al., 1994; Pascual-Marqui, 1999) available in Brain Vision Analyzer 2.1 software (Brain Products, Gilching Germany). LORETA is a discrete, linear, minimum norm, inverse solution that solves the inverse problem by computing the smoothest distribution of current density at each of 2,394 voxels distributed throughout the grey matter and hippocampus within the 3D Montreal Neurological Institute (MNI) brain space (spatial resolution 7 mm). In this manner, a single solution is yielded for a given configuration of the scalp topography at each moment in time.

To determine the brain areas that were active during the significant time windows in the ERPs, statistical analysis focused on the differences in mean source current density solution points within areas of interest that were found to be associated with the processing of dominance in previous fMRI studies. Based on a review of this literature by Watanabe and Yamamoto (2015), the ventromedial and the lateral prefrontal cortex, frontal region, the limbic region, and the cingulate were identified as areas responsive to dominance perception. Consequently, activation in the following ROIs were investigated: the limbic ROI, defined as Brodman’s areas (BA) 20, 28, 34, 36, and 38, bilaterally; the ventro-lateral prefrontal cortex (VLPFC), which comprised BA 10 and 11; and the cingulate ROI, which comprised BA 24 and 32.

The mean source waveforms (in μA/mm^2^) were averaged across voxels in each of the 3 ROIs, for the time windows of interest (N170/VPP: 145-165 ms; P2: 200-220 ms; LPP: 350-430 ms). These values were then extracted and submitted to a three-way ANOVA for repeated measures. Significant effects were further compared using post-hoc Bonferroni comparisons.

## Results

### Performance analysis

Mean reaction times (RTs) were computed for manual responses during the initial part of the experiment, before any explicit ranking, and in the later part of the experiment, after the players were ranked. RTs for the four experimental conditions were the following: inferior players before the rank update: 275 ms (SD = 33.27); inferior players after the rank update: 277 ms (SD = 37.95); superior players before the rank update: 277 ms (SD = 40.29); superior players after the rank update: 284 ms (SD = 78.66).

A 2 (time: pre vs. post ranking) x 2 (rank: inferior vs. superior) repeated measures ANOVA was conducted to determine if the differences in RT were significant. No significant effects were observed either for the main effects of time (F_(1,27)_ = 0.59; *p* = 0.45; h^2^ = 0.006) or rank (F_(1,27)_ = 0.3; *p* = 0.59; h^2^ = 0.006). The interaction was not significant (F_(1,27)_ = 0.12; *p* = 0.73; h^2^ < 0.001).

### ERP analysis

ERPs were computed on artefact-free epochs. After rejection of trials with eye-blinks and noise, an average of 77%, 80%, and 78% of the trials remained available in the baseline, dominant, and nondominant conditions respectively. The number of artefact-free trials did not differ across these three conditions (F(2, 81) = 0.22, *p* = 0.8).

#### P1

A 3 (condition: baseline, dominant, nondominant) x 2 (hemisphere) ANOVA revealed a main effect of Hemisphere, *F*(1, 27) = 18.15, *p* < 0.001, η^2^ = 0.40. The P1 was stronger for the right (6.07 ± 0.682) compared with the left (4.218 ± 0.629) hemisphere, but no other effects were significant.

#### N170

**Fig. 3:**
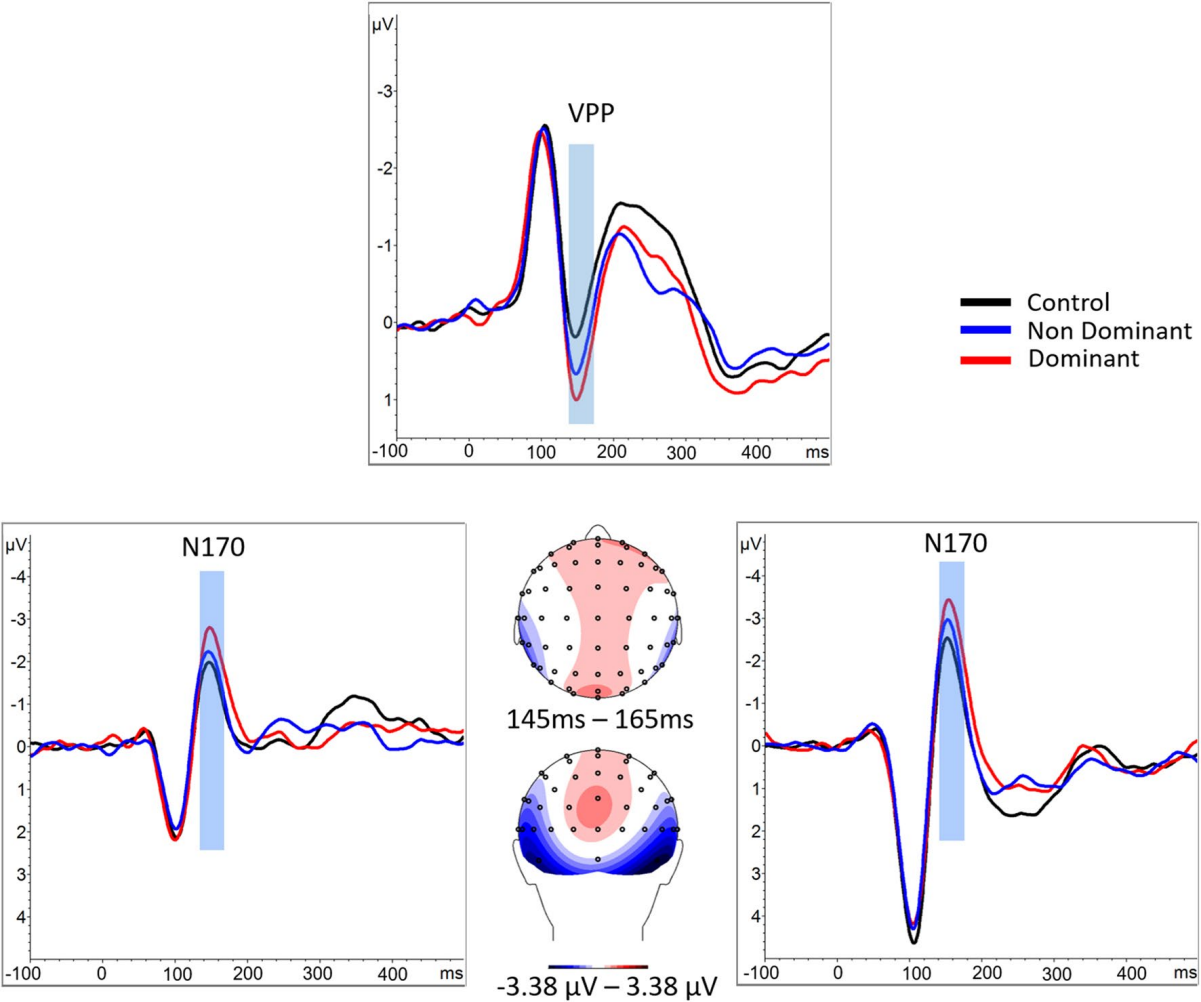
ERPs for control condition (black), dominant (red), and nondominant faces (blue) averaged over the electrodes of interest for the N170 and VPP. The blue boxes highlight the period corresponding to the N170/VPP situated between 145-165 ms. Centre: Scalp topography during the 145- to 165-ms time period viewed from above (top image) and from behind (bottom image) for the mean of the three conditions

A 3 x 2 ANOVA revealed a main effect of condition, *F*(2, 54) = 14.15, *p* < 0.001, η^2^ = 0.34. Focused-cell contrasts revealed that dominant faces (−2.913 ± 0.445) produced a stronger N170 compared with nondominant faces (−2.395 ± 0.437) and the control condition (−2.05 ± 0.456), *Fs*(1, 27) > 10.47, *ps* ≤ 0.003, η^2^ ≥ 0.27. The nondominant faces also produced a stronger N170 compared with the control condition *F*(1, 27) = 5.57, *p* = 0.025, η^2^ = 0.17 (Fig. 3).

#### VPP

When analysing the VPP, a three-way ANOVA using condition as the main effect revealed a main effect of Dominance, *F*(2, 54) = 9.82, *p* < 0.001, η^2^ = 0.27. Focused-cell contrasts revealed that dominant faces (0.849 ± 0.325) produced a stronger VPP compared with nondominant faces (0.515 ± 0.339) and the control condition (0.022 ± 0.375), *Fs*(1, 27) > 4.97, *ps* ≤ 0.034, η^2^ > 0.15. The nondominant faces also produced a stronger VPP compared with the control condition *F*(1, 27) = 5.12, *p* = 0.032, η^2^ = 0.16.

#### P2

**Fig. 4:**
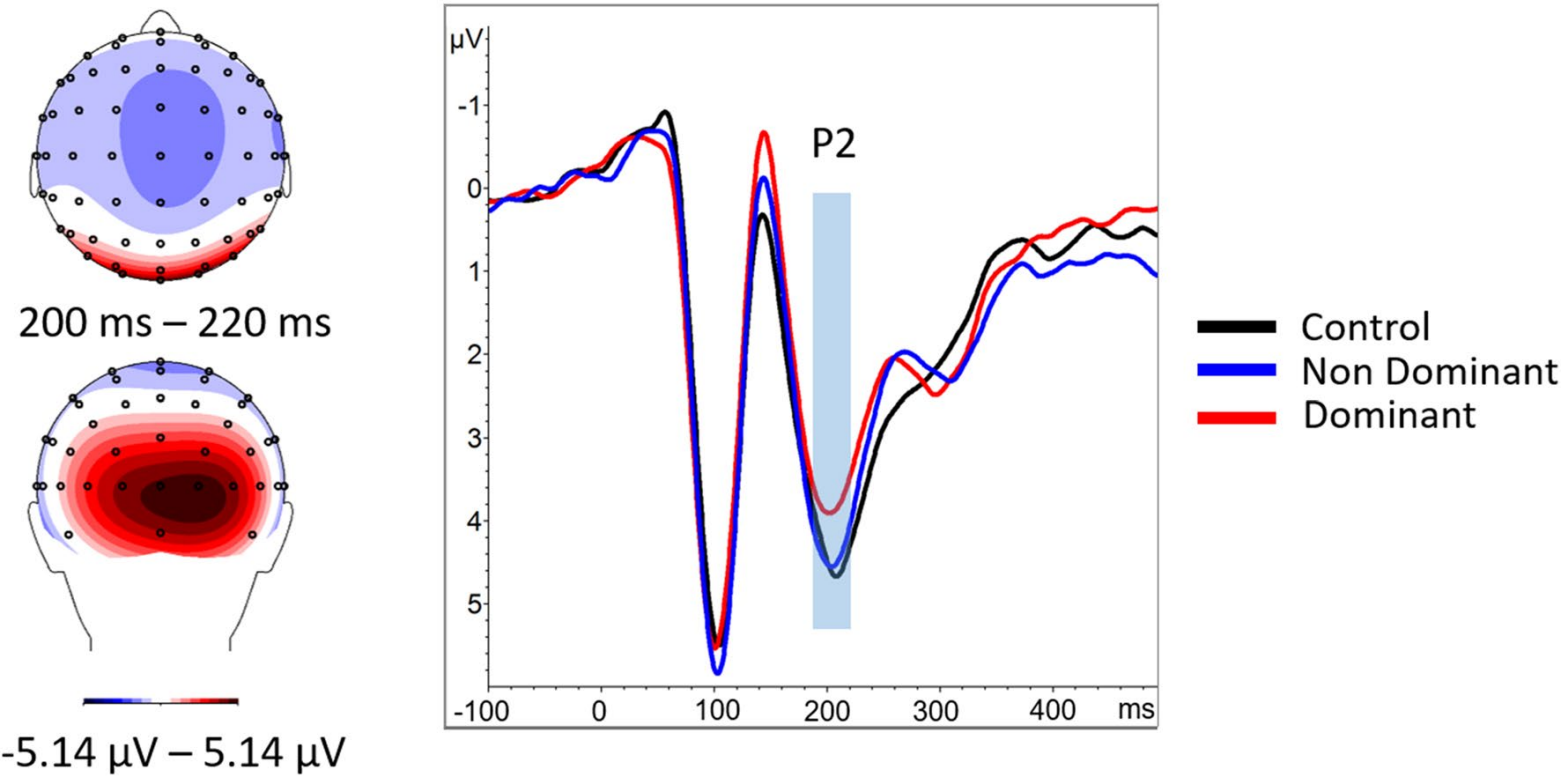
Right: ERPs for control condition (black), dominant (red), and nondominant faces (blue) averaged over the electrodes of interest for the P2 (electrodes Iz, O1, O2, Oz, PO7, and PO8). The period for the P2 is highlighted with the red box. Left: Mean P2 scalp topography viewed from above (top left) and behind (bottom left) for the three conditions averaged during the P2 time window

The three-way ANOVA using condition as the main effect revealed a significant difference, *F*(2, 54) = 4.66, *p* = 0.014, η2 = 0.15. Focused-cell contrasts showed that dominant faces (3.79 ± 0.637) produced a lower P2 compared with nondominant faces (4.431 ± 0.597) and the control condition (4.573 ± 0.602), *Fs*(1, 27) > 5.41, *ps* ≤ 0.028, η^2^ > 0.16. The nondominant faces did not significantly differ from the baseline, *p* = 0.566 (Fig. 4).

#### LPP

**Fig. 5:**
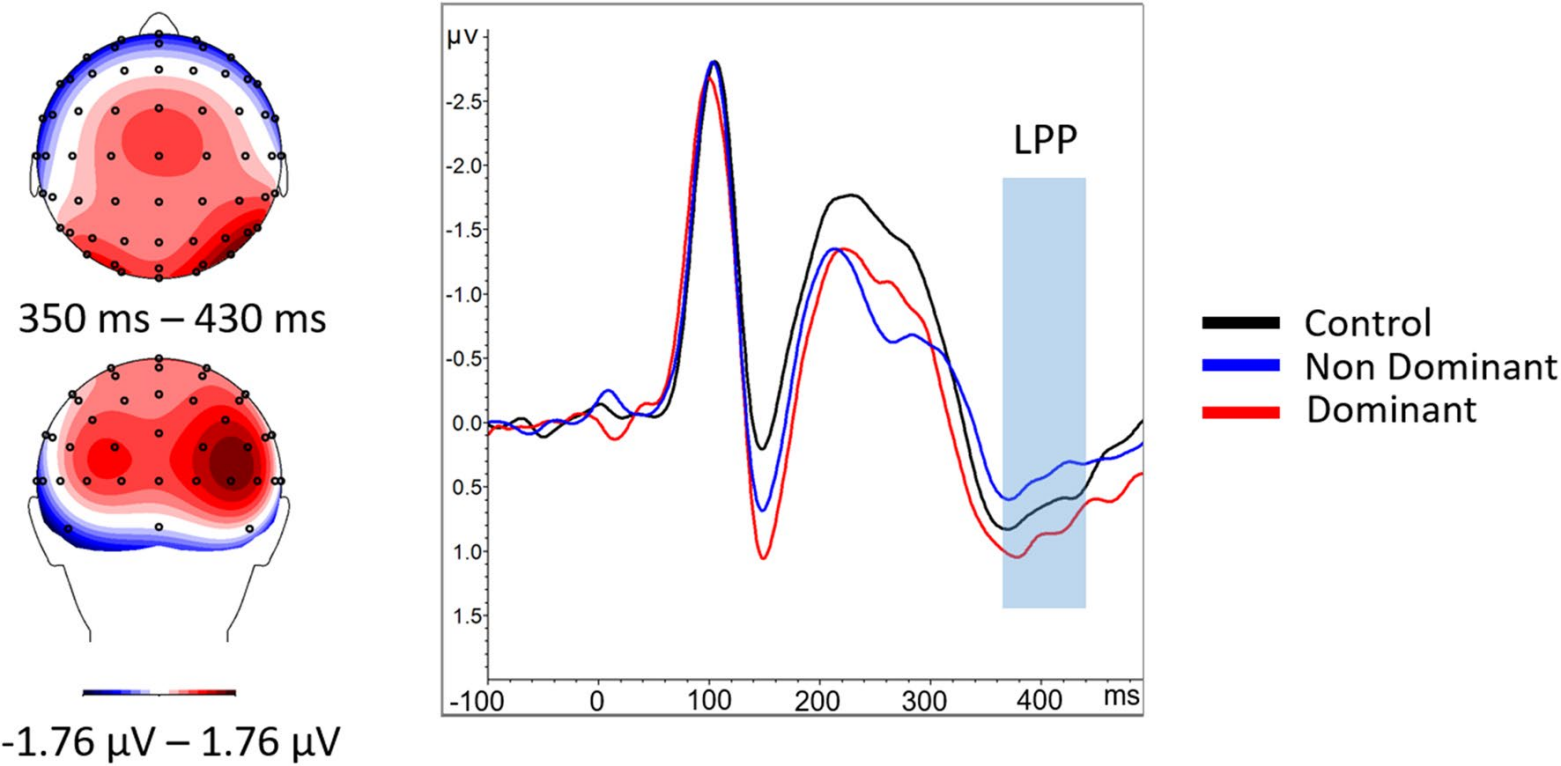
Right: ERPs for control condition (black), dominant (red) and nondominant faces (blue) averaged over the electrodes of interest for the LPP (electrodes C1, C2, Cz, CP1, CP2, CPz, FC1, FC2, FCz ). The LPP is highlighted in red. Left: Mean scalp topographies viewed from above (top left) and behind (bottom left) for the three conditions averaged during the 350 to 430 ms time window

The three-way ANOVA revealed a main effect of condition, *F*(2, 54) = 3.19, *p* = 0.049, η^2^ = 0.11. Focused-cell contrasts revealed that dominant faces (0.919 ± 0.342) produced a stronger LPP compared with the nondominant faces (0.453 ± 0.309), *Fs*(1, 27) = 7.48, *ps* = 0.011, η^2^ = 0.22 (Fig. 5). No other significant effects were found (*ps* > 0.161).

### Source localization analysis

#### N170 (145-165ms time period)

The LORETA solutions in the Limbic ROI, comparing dominant, nondominant, and baseline conditions revealed a main effect of condition, *F*(2, 54) = 5.53, *p* = 0.007, η^2^ = 0.17. Focused-cell Bonferroni tests revealed that dominant faces (0.00293 ± 0.00023) produced a significantly greater activation compared to both nondominant faces (0.00231 ± 0.00023) and baseline conditions (0.00213 ± 0.00020; both *ps* < 0.039), whereas the nondominant and baseline conditions did not significantly differ (*p* = 1). The three-way ANOVAs for the VLPFC and Cingulate ROIs were nonsignificant (*ps* > 0.138).

#### P2 (200-220-ms time period)

The three-way ANOVA for the limbic region revealed a main effect of condition, *F*(2, 54) = 4.55, *p* = 0.015, η^2^ = 0.14. Focused-cell Bonferroni tests revealed that dominant faces (0.00299 ± 0.00027) produced a significantly (*p* = 0.017) stronger activation compared with nondominant faces (0.00237 ± 0.00025). The baseline condition (0.00284 ± 0.00032) did not significantly differ from the dominant and nondominant faces conditions (*ps* > 0.09). The three-way ANOVAs for the VLPFC and Cingulate ROIs were nonsignificant (*ps* > 0.188).

**Fig. 6:**
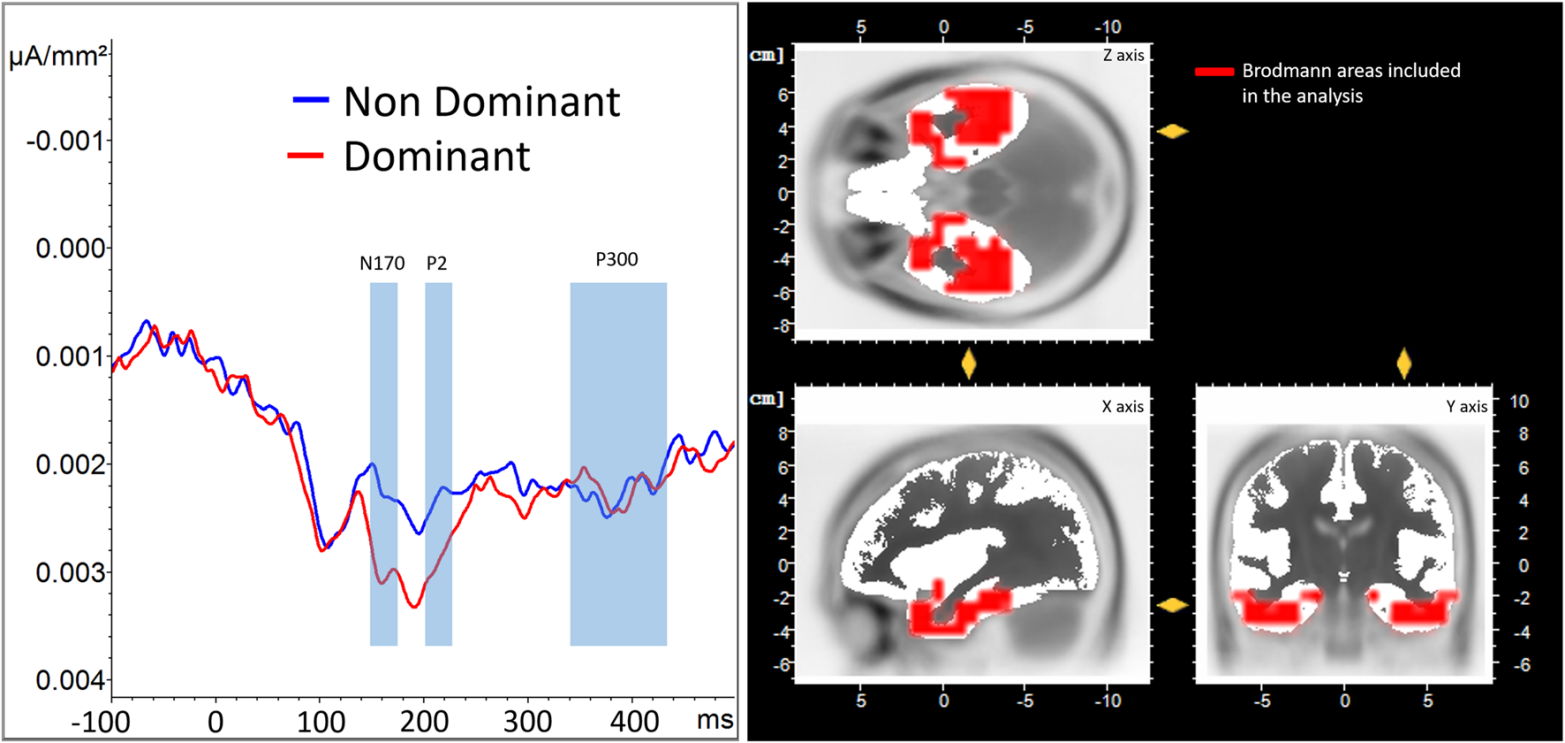
*Left panel*: mean current density (y-axis in uV/mm^2^) over time (x-axis in ms) for dominant (red line) and nondominant faces (blue line) in the Limbic ROI. Blue boxes indicate the time periods corresponding to the N170, P2, and LPP periods identified in the ERP analyses. *Right panel:* Overlay of the ROI (highlighted in red), superimposed on a horizontal (top), sagittal (bottom left) and coronal (bottom right) sections of the MNI average brain. Mean activity in this ROI was significantly greater for dominant faces during the N170 time window (yellow pointers indicate location of maximum activity in this ROI during this window, and correspond to MNI coordinates: X = 39, Y = −7, Z = −24)

Figure 6 (left) plots the mean current source density values in the Limbic ROI across the entire epoch (−100 ms to 500 ms), for dominant and nondominant faces. The three periods of interest (N170, P2, and LPP) are highlighted with colored boxes. In the right panel, the voxels within the Limbic ROI, which are significantly more activated during the N170 and P2 periods, appear in red, superimposed on the average MNI brain.

#### LPP (350-430-ms time period)

Comparisons of the mean source current density between dominant and nondominant conditions did not reach the 0.05 threshold in either of the three ROIs retained in our initial hypothesis. Consequently, we decided to adopt a data-driven approach by identifying the areas activated during the LPP time window and subsequently comparing them statistically.

**Fig. 7:**
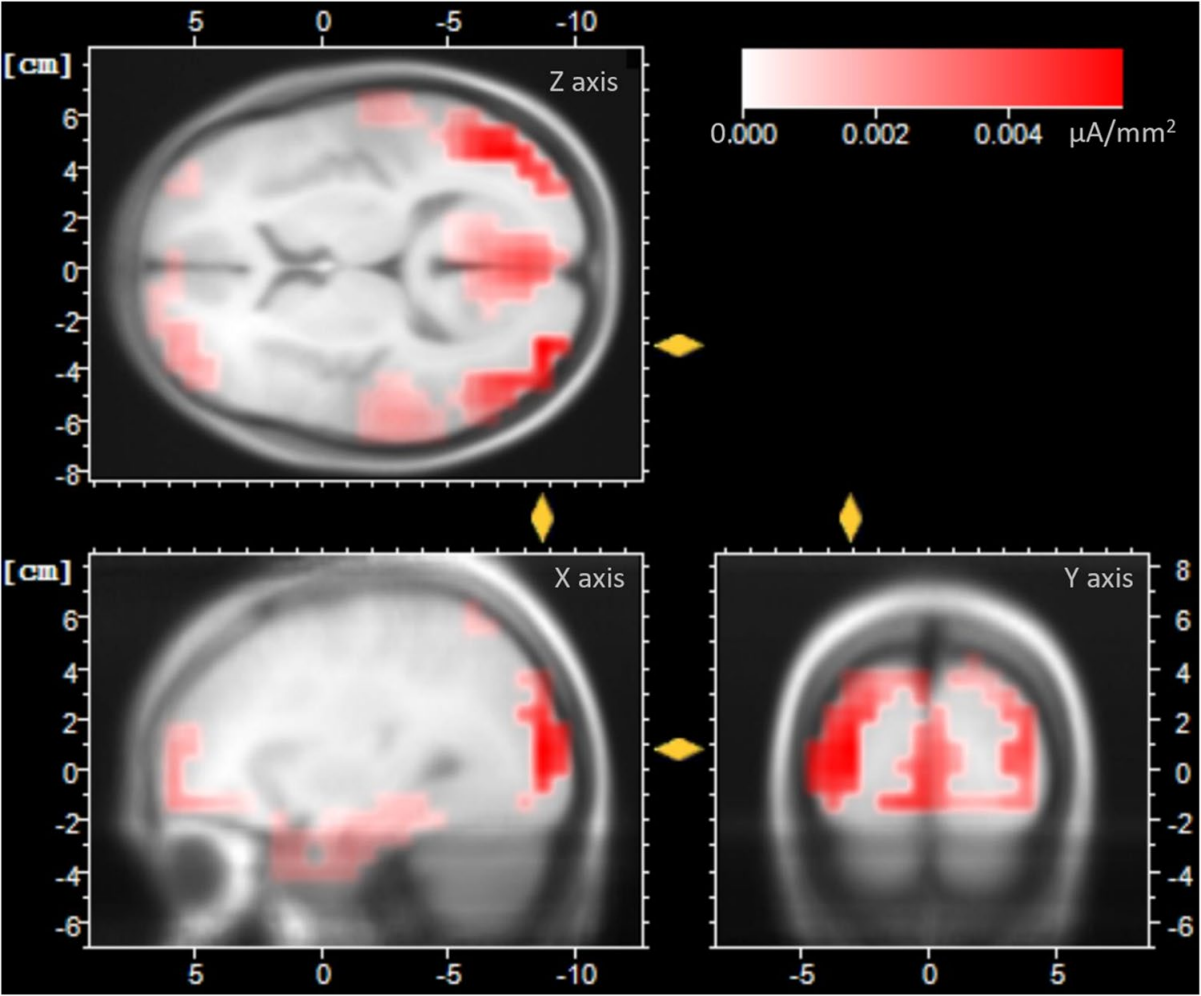
Mean current density during the LPP time period (350-430 ms) for faces averaged across all conditions. The scalp topography of the LPP was best explained by activity in the extrastriate visual cortices (BA 19), as well as anterior temporal (BA 20, BA 37, BA38) and prefrontal areas (BA 10, BA 11)

The grand mean ERP of all conditions was therefore used to visualize the sources activated during the 350-430-ms time window (Fig. 7). Regions of greater activation were observed in visual extrastriate areas and (BA 19), anterior temporal regions (BA 20, 37, 38), in addition to the anterior regions that had been part of our initial hypothesis (BA 10, 11).

To ascertain whether the visual and temporal cortices differed significantly across conditions of dominance, statistical comparison were further computed for the ROIs composed of BA 19 and of BA 20, 37, and 38, but neither reached the 0.05 threshold of significance.

## Discussion

In this study, naïve participants carried out a speeded reaction time procedure, which was manipulated so that the other (fictitious) players were seen as either more, or less efficient at the task, thus producing a sense of hierarchy. ERPs were measured when participants viewed the faces of the players who ranked higher (dominant players) or lower (non-dominant) than them during the task. The brain response to these faces differed as a function of hierarchy already at around 170 ms, during the so-called N170 component which is generally described as a face-sensitive component (Bentin et al., 1996; Itier & Taylor, 2004). Greater amplitudes were observed for individuals who became rapidly dominant during the game, compared to those who were less dominant, supporting evidence that social hierarchy modulates the early stages of face processing. In addition, the amplitude of the late positive potential (LPP), occurring between 400–700 ms, also was found to differ according to the social rank of the face, with an enhanced LPP observed when participants viewed the faces of dominant players.

Interestingly, two previous studies (Feng et al., 2015; Santamaría-García et al., 2015) found N170 modulations for images of dominant players, using a different procedure. In this case, participants were asked to estimate rapidly the number of dots in an array. They were made to believe that some players were better or worse than them at this task. In line with our current findings, the ERP response to the faces of superior players also was found to enhance the N170, suggesting that the judgment of social hierarchy occurs rapidly when viewing anonymous conspecifics.

However, early processing of dominant faces has not been reported systematically. For example, Breton et al. (2014) asked their participants to carry out a dot-evaluation task and subsequently presented photographs of the other alleged players, asking them to recall the hierarchical status of the players (superior, similar, or inferior). The authors found no modulation of the N170 in response to dominant faces, but observed an enhanced LPP between 400-700 ms, consistent with the results found on the LPP of our current experiment. They suggested that the processing of social hierarchy was a later, more elaborate cognitive process. Breton et al. (2019) further confirmed their findings in a subsequent study. In this experiment, the authors presented faces of fictional individuals along with their associated high or low-income job titles. After associating each individual with a social category, the ERPs were again measured. Again, the authors did not find any significant effects on the N170 but reported a modulation of the LPP according to the social rank of the face, with greater positivity evoked by dominant faces.

The lack of consensus regarding the effects of dominance on the N170 may be due to the differences in procedure. Indeed, the studies by Feng et al. (2015) and Santamaría-García et al. (2015), which both reported N170 modulation, provided hierarchical cues with the faces, whereas the paradigms used by Breton et al. (2019), which found only the later LPP effect, used association tasks in which faces had to be remembered for subsequent EEG recording.

This raises the question of whether in our experiment, the stars presented with the face stimuli may have influenced the ERP response. However, this possibility is precluded by the absence of any early ERP modulation, in particular the absence of a P1 modulation linked to dominance. Moreover, this possibility was also refuted by Feng et al. (2015), who noted that in their related study, when the stars were present, but not indicative of dominance, the N170 did not show a similar modulation.

The effect of dominance on the N170 can be interpreted in the light of previous observations in the field of emotion processing. Many studies, although not all, have reported N170 modulations for a range of emotional facial expressions (Batty & Taylor, 2003; Blau et al., 2007; see Schindler & Bublatzky, 2020 for a recent review), with the highest effects observed for angry and fearful expressions. It has been argued that the modulation of the N170 may be the result of activation of a subcortical pathway to the amygdala by the face stimuli (e.g., Pegna et al., 2008). Indeed, one of the roles of this pathway would be to enhance visual processing of salient stimuli, such as fearful or angry faces. This idea was addressed by an fMRI study (Vuilleumier et al., 2004) that compared the fMRI response in extrastriate visual areas to fearful and neutral faces in patients with hippocampal sclerosis that included or excluded the amygdala. Patients with intact amygdala showed an enhanced extrastriate visual response to fearful faces compared with neutral faces, whereas no such difference was observed in patients with amygdala sclerosis, leading to the conclusion that the amygdala was responsible for the increased visual extrastriate activity for emotional faces, likely through retrograde feedback projections to extrastriate regions. Recently, the likelihood that the N170 enhancement for emotional faces may rely on the amygdala was confirmed by Framorando et al. (2021), who found that patients with resection of the (right) amygdala, failed to show the expected 170 enhancement for fearful faces.

Similar effects were found on the N170 and VPP. Since both coincided in their maximum occurrence, these components were taken to reflect the same scalp topography. As noted above, the N170 has been hypothesized to be the positive counterpart of the N170 (Joyce & Rossion, 2005), and as such it is plausible to assume that any conclusions drawn for the N170 can be drawn for the VPP. Moreover, insofar as the same topography reflected both components, the results of the inverse solution were necessarily identical whether considering the N170 or the VPP.

With this in mind, the LORETA source localisation algorithm in our current study showed a greater activation in the limbic region—a large area that includes the amygdala—for dominant faces, corroborating the idea that the enhanced N170 is due to an early amygdala responsiveness for faces whose salience is determined by social dominance.

The role of the amygdala on the processing of social dominance has been highlighted with other approaches. In rhesus monkeys, neonatal resection of the amygdala and hippocampus was seen to alter dominant behaviour, causing them to rank lower on all indices of social dominance (Bauman et al., 2006). Functional MRI studies in humans revealed modulation of amygdala activity in response to social dominance, especially when social hierarchy was not fixed (Zink et al., 2008). These authors examined BOLD signals in the amygdala in response to dominance through a virtual game using a star ranking similar to the one used in the current investigation. In their experiment, participants could play against a superior or inferior player in the context of a stable social hierarchy (i.e., the participant’s rank did not change during the game) or an unstable one in which the participant's rank could be modified by the outcome of the game. The amygdala showed increased activity to the viewing of superior players when the game involved an unstable hierarchy. The authors suggested that in unstable hierarchies, a sense of loss of control and predictability ensues, resulting in high levels of physical stressors (Sapolsky, 2004, 2005), which affects the perception of the dominant individual.

It is therefore likely that the increased N170/VPP activity reported in the current study may be linked to amygdala activation in response to a dominant individual. Social hierarchy appears to engage the amygdala early in the stream of visual processing, due to its relevance and emotional significance.

A significant effect also was observed on the subsequent P2 component, characterized by a decrease in P2 amplitude for dominant faces. A number of studies have reported changes in P2 amplitude in tasks involving emotional stimuli and have suggested that this may result from modulations in attentional deployment (Carretié et al., 2004; Carretié et al., 2013; Holmes et al., 2006; Kanske et al., 2011). Here, enhancements of the anterior P2 were seen to reflect greater attention. However, others have suggested that a decreased P2 may be observed for salient stimuli. In one study (Straube & Fahle, 2010), the saliency of a figure embedded in a matrix of Gabor elements was systematically manipulated. The results showed that the posterior P2 amplitude, occurring at around 180 to 250 ms, was significantly attenuated when stimulus saliency was increased. A recent study by Yu et al. (2022) showed a similar P2 decrease for looming angry faces, which was interpreted as an attentional modulation in response to a more salient stimulus. Our observations are supported by these findings. Indeed, we would conjecture that socially dominant faces present a greater saliency, giving rise to the P2 modulation. The generators associated with this time window, being also situated in the limbic region, appear to further corroborate this interpretation hypothesis since previous evidence has shown that the amygdala is involved in the processing of salience (Santos et al., 2011).

The results of the LPP followed a pattern that was similar to the N170/VPP with a stronger response for the hierarchical dominant faces compared to the non-dominant and control conditions. LPP enhancement was already observed in four separate studies examining the ERP response to faces of hierarchically superior individuals based on performance (Breton et al., 2014; Feng et al., 2015; Furley et al., 2017; Breton et al., 2019). Interestingly, a separate study reported a modulation of LPP by hierarchies based on wealth, where the view of high social status individuals elicited a greater P300 (which bears some resemblance to the LPP; Hajcak & Foti, 2020) than that of low social status individuals (Gyurovski et al., 2018). Overall, the enhanced LPP triggered by faces of individuals occupying a higher hierarchical status thus appears to be a robust phenomenon.

It has been claimed that the LPP is linked to the motivational significance of a stimulus. For example, sexual stimuli, money, or appetitive stimuli, which can be considered motivationally relevant, have been reported to elicit greater LPP compared to control stimuli (Briggs & Martin, 2009; Gable & Harmon-Jones, 2010). The LPP thus has been interpreted as resulting from sustained and elaborate processing of significant stimuli (Bradley et al., 2007; Flaisch & Schupp, 2013), and in particular the activation of attentional and motivational systems (Lang & Bradley, 2010; Sapolsky, 2005; Schupp et al., 2000).

In the current context, the enhanced LPP found for dominant faces therefore appears attributable to the enhanced attention and increase in motivational resources that result from the imagined interaction with dominant individuals (Foulsham et al., 2010; Ratcliff et al., 2011).

Attempts to identify the structures responsible for the LPP have been made using EEG and fMRI paradigms during the viewing of emotional images (Liu et al., 2012; Sabatinelli et al., 2013). The results of these investigations showed that visual, temporal, prefrontal (orbitofrontal), and amygdala areas were correlated with LPP amplitude. It was concluded that the LPP was generated by an extensive brain network, which includes subcortical and cortical brain structures. In the field of emotion processing, this later, broader cortical activation has been posited to reflect a second, more detailed, and elaborate stage of processing stage, which would necessarily encompass a larger number of cortical areas (Schupp et al., 2006).

In our investigation, no statistically significant differences were found in the source localisation results; however, significant ERP differences were seen for social dominant face stimuli during the N170/VPP and LPP. Based on the latter results, it appears that, as for emotion processing, the processing of socially hierarchical status occurs within two distinct time periods. An initial, rapid activation occurs initially around 170 ms involving rapid perceptual processes and activation of limbic areas. This is followed by a second, slightly later process arising after 350 ms that involves motivational and attentional systems and likely engages a larger neural network (Schupp et al., 2006).

A burgeoning area research has identified dominance via sex differences in facial morphology (Dixson, 2022), facial structure (Caton et al., 2022a, b; Mefodeva et al., 2020), gender (Wang et al., 2019), and facial hair (Dixson et al., 2017; Sherlock et al., 2017; Dixson & Vasey, 2012), as well as the interaction between beardedness and facial expressions (Craig et al., 2019; Dixson et al., 2021, 2022). Such observations suggest that male faces may be perceived as more threatening in general than female faces (Mazurski et al., 1996; Öhman & Dimberg, 1978; Kret & Gelder, 2012). Our current sample does not allow the analyses of potential gender effects, however future research may explore more closely the influence of facial morphology within and between-sexes on judgments of social dominance.

## Data Availability

The experiment reported in this manuscript was not preregistered. The datasets generated and analysed during the current study are available upon request to the corresponding author.
